# Nonalcoholic fatty liver disease – a growing public health problem

**DOI:** 10.3325/cmj.2021.62.1

**Published:** 2021-02

**Authors:** Ivana Mikolašević, Tajana Filipec Kanižaj, Giovanni Targher

**Affiliations:** 1 Department of Gastroenterology, University Hospital Center Rijeka, Rijeka, Croatia; 2 Department of Gastroenterology, Merkur University Hospital, Zagreb, Croatia; 3 Faculty of Medicine, University of Rijeka, Rijeka, Croatia *ivana.mikolasevic@gmail.com*; 4 Zagreb University School of Medicine, Zagreb, Croatia; 5 Section of Endocrinology, Diabetes, and Metabolism, Department of Medicine, University of Verona, Verona, Italy

Over the past years, the global burden of chronic liver diseases (CLDs) has been steadily increasing, irrespective of age, sex, region, or race. The European Union (EU) countries have the highest CLDs burden in the world, with almost 30 million people suffering from CLDs (1,2). Unrecognized and often untreated, CLDs may progress to more advanced stages, such as cirrhosis, liver failure, and hepatocellular carcinoma (HCC). Global and country-specific estimates of the disability-adjusted life years and years of life lost place cirrhosis within the top 20 causes (1).

In EU countries, the most common causes of cirrhosis and the most frequent indications for liver transplantation in 2013 were alcoholic liver disease (ALD, 25%-45%), chronic hepatitis C (HCV, 30%-35%), and chronic hepatitis B (HBV, 10%-20%) (3). However, with the implementation of prevention, screening, and treatment (direct antiviral agents) programs for chronic viral hepatitis, in most countries ALD and nonalcoholic fatty liver disease (NAFLD) have overtaken viral hepatitis as the primary causes of cirrhosis (4). Over half a million individuals worldwide every year develop incident HCC. The global incidence of this liver cancer has been steadily rising, making HCC the fifth most frequently diagnosed cancer (3,5). NAFLD is also the most rapidly growing cause of HCC among US patients listed for liver transplantation (6). Similarly, NAFLD is the fastest growing cause of HCC in liver transplant candidates both in the European Liver Transplant Registry and in the United Network for Organ Sharing databases (7-9). Many authors believe that NAFLD will in the foreseeable future overtake ALD as the leading indication for liver transplantation in CLD patients. This finding is not surprising, because today NAFLD is the most common cause of CLD worldwide, and its prevalence parallels the increasing global prevalence of obesity, metabolic syndrome (MetS), and type 2 diabetes (T2DM) (10). An updated meta-analysis of 86 observational studies from 22 countries (involving more than 8.5 million individuals) reported that the global prevalence of NAFLD in the general adult population was around 25%, with the highest prevalence in the Middle East and South America (11). NAFLD is also strongly associated with MetS and its individual components, such as central obesity, T2DM, hypertension, and atherogenic dyslipidemia. NAFLD, increasingly referred to as the liver manifestation of the MetS (10), is a metabolic liver disease that encompasses a pathological spectrum of progressive liver conditions ranging from simple steatosis to nonalcoholic steatohepatitis, cirrhosis and, ultimately, HCC (12). One of the largest cohort studies on the clinical course and progression of NAFLD conducted in Sweden has reported that the liver fibrosis stage is the strongest histologic risk factor for liver-related morbidity and mortality in NAFLD (13). Thus, we need patient-friendly, easy-to-use, and inexpensive non-invasive tests for the detection of significant and advanced liver fibrosis.

A further important clinical problem when it comes to NAFLD is that HCC may develop also in patients with non-cirrhotic NAFLD. In fact, some studies demonstrated that HCC could develop in NAFLD patients who do not have cirrhosis, especially in those with nonalcoholic steatohepatitis with or without fibrosis. Thus, HCC occurrence in non-cirrhotic NAFLD is one of the most worrisome aspects of HCC in NAFLD (14). HCC is also the most overlooked complication in patients with NAFLD and the most challenging issue in everyday clinical practice in this patient population (14). Currently, effective screening programs are largely limited due to incomplete understanding of liver carcinogenesis in NAFLD patients, as well as due to lack of diagnostic methods that can stratify the HCC risk in patients with NAFLD. However, given the epidemic proportions of NAFLD in the general population worldwide, HCC screening in all people with NAFLD (especially in those without cirrhosis) is unfeasible. Consequently, poor surveillance relates to an often-limited treatment options for NAFLD-related HCC (14). As it has been discussed above, there are several open questions about HCC in NAFLD, such as the timing of carcinogenesis in non-cirrhotic patients with NAFLD and the best diagnostic approaches that will detect high-risk patients (14). Worryingly, NAFLD is a growing cause of CLDs also in the pediatric population. The pediatric population with NAFLD will face an increased risk of liver-related morbidity and mortality in adulthood (14). These observations indicate the need for a global policy for the prevention of obesity and its chronic complications starting from childhood (14).

Another important issue in the context of NAFLD is its strong association with the risk of many extrahepatic diseases. In the past decade, strong evidence has been provided of adverse effects of NAFLD extending beyond the liver, and of NAFLD being not just a liver disease but a multisystem disease (15). Cardiovascular diseases are a well documented predominant cause of death in patients with NAFLD (16). However, growing evidence also indicates that NAFLD is associated with an increased risk of developing T2DM and chronic kidney disease (17-21), as well as some other chronic diseases, such as polycystic ovary syndrome, psoriasis, obstructive sleep apnea, and some types of extra-hepatic malignancies (eg, colorectal and breast cancers) (12,15,17). These associations could be simply a consequence of the shared cardiometabolic risk factors (ie, insulin resistance, MetS and its individual components). A growing body of evidence, however, suggests that NAFLD is related to many of these extra-hepatic diseases independently of the shared cardiometabolic risk factors. Another interesting finding is that in some cases the association between NAFLD and extra-hepatic diseases is bidirectional (12,15,17). For example, NAFLD is not only a simple consequence of T2DM but may also act as a causal risk factor for T2DM. Indeed, two large meta-analyses confirmed that NAFLD was associated with a ~ 2.2-fold increased risk of incident T2DM, and that this risk parallels the underlying severity of NAFLD, especially the severity of liver fibrosis (12,20). On the other hand, in T2DM patients NAFLD worsens the glycemic control and is associated with an increased risk of all-cause mortality (12). Accordingly, the coexistence of T2DM and NAFLD is not only associated with the risk of NAFLD progression, but also with the risk of chronic vascular complications of diabetes (12,22). Based on all these considerations, all patients with NAFLD would benefit from a periodical screening for T2DM, cardiovascular diseases, and chronic kidney disease (15). Despite the growing evidence that NAFLD, especially its more severe forms (ie, advanced fibrosis), are related to the risk of cardiovascular diseases and other extra-hepatic diseases, large prospective and mechanistic studies are needed to confirm that NAFLD is actively involved in the pathophysiology of specific extrahepatic chronic diseases in different NAFLD populations (15). In addition, further research is needed to address the cost-effectiveness of screening for extrahepatic diseases in all patients with NAFLD.

While significant progress has been made in the prevention and pharmacological treatments of some CLDs in many parts of the world (eg, with introduction of direct-acting-drugs for HCV, vaccination for HBV, behavioral changes for ALD), prevention, screening, and effective treatment of NAFLD has not yet been achieved globally. Knowing that most CLDs, including NAFLD, are preventable and treatable, the urgent need arises for action plans on preventive measures, screening, and pharmacological treatment options of this very common and burdensome liver disease.
